# Assessing the Impact of Aerial Pesticide Spraying: Mancozeb Exposures among Pregnant Women Living near Banana Plantations

**DOI:** 10.1289/ehp.122-A337

**Published:** 2014-12-01

**Authors:** Carol Potera

**Affiliations:** Carol Potera, based in Montana, also writes for *Microbe*, *Genetic Engineering News*, and the *American Journal of Nursing*.

Mancozeb is sprayed on bananas to prevent black sigatoka, a fungal disease that impairs fruit ripening and reduces banana yields.^1^ In this issue of *EHP*, investigators report that pregnant women living near banana plantations in Costa Rica have elevated urinary levels of ethylene thiourea (ETU), a metabolite of mancozeb.^2^ At 2.9 µg/L,^2^ the median ETU level in these women was nearly twice the 95th percentile of exposure measured in pregnant women in California who lived near ground spraying of mancozeb.^3^

**Figure:**
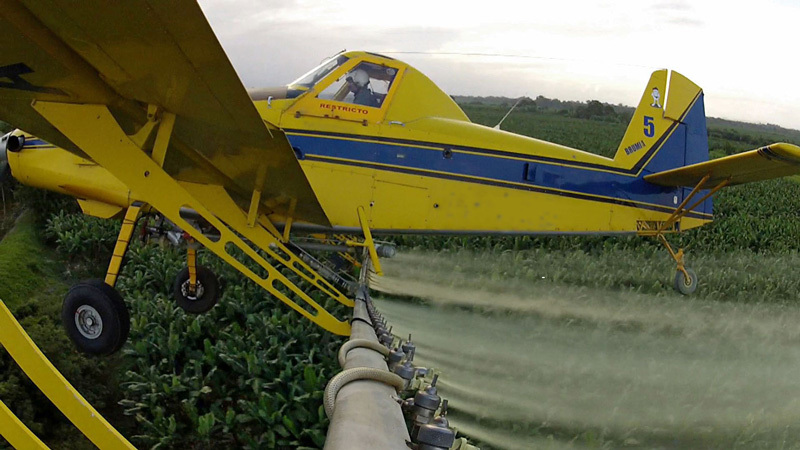
New findings suggest that current regulations governing aerial application of pesticides may not adequately protect pregnant women living near Costa Rican banana plantations. © Marcus Winterbauer, WDR/Längengrad Filmproduktion GmbH

For the current study, researchers analyzed urine samples collected from 445 pregnant women enrolled in the Infants’ Environmental Health Study in Limón, Costa Rica. All the women lived within 5 km of a banana plantation. Three-quarters of them were housewives or did not work, while 8% (plus 63% of their partners) reporting working on a banana plantation or performing other agricultural work. None reported applying mancozeb herself.

Urine samples were collected up to three times during pregnancy. The highest ETU levels occurred in women living within 50 m of a banana plantation. These women had urinary ETU concentrations about 45% higher than the women who lived farthest away. ETU concentrations also were elevated in pregnant women who had recently washed farmworkers’ clothes and in those who worked in banana packing plants. Nearly three-fourths of the women had an estimated daily intake of ETU exceeding the reference dose of 0.08 µg/kg/day^4^ set by the U.S. Environmental Protection Agency for chronic oral exposure.^2^

The researchers are currently analyzing data they collected on the women’s babies, including gestational age at birth, growth, and neurodevelopment at 12 months of age. “We also are in the process of seeking funds to evaluate growth, behavior, and respiratory symptoms at age four years and a later date,” says study leader Berna van Wendel de Joode, a professor at National University’s Central American Institute for Studies of Toxic Substances in Heredia, Costa Rica.

Animal studies show that ETU, at high concentrations, interferes with fetal brain cell development^5^ and disrupts thyroid function after chronic exposure to lower concentrations.^6^ Proper levels of thyroid hormones are needed to regulate fetal brain development.^7^ Mancozeb has been associated with hypothyroidism in nonpregnant women exposed agriculturally in Iowa and North Carolina.^8^

For the pregnant Costa Rican women, “the relatively high levels of ETU pose a theoretical risk to their fetuses,” says endocrinologist Whitney Goldner of Nebraska Medicine in Omaha; if exposure were to result in untreated hypothyroidism during pregnancy, the women’s children would be at increased risk for neurocognitive developmental problems in the future, she says.

Aerial spraying, which occurred weekly in the study area, may increase respiratory and dermal uptake of mancozeb, according to the authors. They recommend that aerial application of pesticides should be reduced and/or replaced with more easily controlled methods; homes should be built farther from banana plantations with vegetative barriers planted between them; and work clothes should be machine-washed at the workplace rather than hand-washed at home.^2^ In future studies, Goldner says, pregnant women with high ETU levels ideally would be screened for hypothyroidism and treated if necessary to prevent any risk to the fetus.

Researchers elsewhere are seeking more sustainable methods for growing bananas. For example, at EARTH University (Escuela de Agricultura de la Región Tropical Húmeda), a research facility and working banana plantation in Costa Rica, a proprietary blend of nontoxic fungi-killing bacteria and yeast replace some of the mancozeb sprayed.^9^ Researchers at CIRAD (French Agricultural Research Center for International Development) in Martinique, French West Indies, have bred a banana hybrid that resists diseases and pests.^10^ “It’s important to do more research on alternative pest control methods,” says van Wendel de Joode.
